# Comparison of SARS-CoV-2 Antibody Response After 2-Dose mRNA-1273 vs BNT162b2 Vaccines in Incrementally Immunosuppressed Patients

**DOI:** 10.1001/jamanetworkopen.2022.11897

**Published:** 2022-05-16

**Authors:** Jonathan Mitchell, Caoilfhionn M. Connolly, Teresa Po-Yu Chiang, Jennifer L. Alejo, William A. Werbel, Dorry L. Segev, Allan B. Massie

**Affiliations:** 1 Department of Surgery, Johns Hopkins University School of Medicine, Baltimore, Maryland; 2 Division of Rheumatology, Department of Medicine, Johns Hopkins University School of Medicine, Baltimore, Maryland; 3 Division of Infectious Diseases, Department of Medicine, Johns Hopkins University School of Medicine, Baltimore, Maryland

## Abstract

This cohort study compares the antispike antibody titers of the mRNA-1273 and BNT162b2 vaccines for SARS-CoV-2 in incrementally immunosuppressed patients.

## Introduction

Both mRNA-1273 and BNT162b2 SARS-CoV-2 vaccines elicit immune responses consistent with viral neutralization in most immunocompetent persons.^1,2^ Immunosuppressed individuals, such as persons with rheumatic and musculoskeletal diseases (RMDs) and solid organ transplant recipients (SOTRs), have decreased immune responses to these vaccines.^3,4^ Thus, differential vaccine immunogenicity is clinically relevant in ways not seen in immunocompetent persons. This study compares antispike antibody titers after the 2-dose mRNA-1273 and BNT162b2 vaccines in incrementally immunosuppressed patients.

## Methods

Patients with RMDs and SOTRs without prior positive SARS-CoV-2 test results were recruited to this cohort study as previously described.^3,4^ Participants receiving 2 SARS-CoV-2 messenger RNA (mRNA) doses between December 16, 2020, and July 6, 2021, were included. This study followed the STROBE reporting guideline for observational studies and was approved by the Johns Hopkins Institutional Review Board. Patients provided informed consent electronically.

Semiquantitative testing for antibodies against the receptor binding domain (RBD) of the SARS-CoV-2 spike protein was performed using the Roche Elecsys anti–SARS-CoV-2 S enzyme immunoassay 15 to 45 days after the second dose. Anti-RBD titers were divided into 50, 100, and 250 U/mL or greater based on levels associated with incremental plasma neutralizing capacity in patients convalescing from COVID-19 and a modeling study across COVID-19 vaccine trials.^5,6^ Participants were stratified by increasing intensity of immunosuppression: patients with RMDs not receiving immunosuppression or receiving hydroxychloroquine or intravenous immunoglobulin, patients with RMDs receiving immunosuppression, SOTRs not receiving mycophenolic acid or mycophenolate mofetil, and SOTRs receiving mycophenolic acid or mycophenolate mofetil. Immunosuppression intensity was defined by the presence or absence of baseline immune dysregulation and data on SARS-CoV-2 vaccination antibody response.^3,4^ We compared response rates between mRNA-1273 and BNT162b2 recipients using modified Poisson regression weighted for age, time since vaccination, and number of immunosuppressive medications and repeated this analysis for positive response thresholds of 50, 100, and 250 U/mL.

Participant characteristics were compared using the Wilcoxon rank sum test for continuous and Fisher exact test for categorical variables. A 2-sided *P* < .05 was considered significant. Analyses were performed using Stata/SE, version 17.0 (StataCorp LLC).

## Results

This cohort included 1158 participants with RMDs and 697 SOTRs: 647 patients with RMDs (55.8%) and 367 SOTRs (52.7%) received BNT162b2; the remainder received mRNA-1273 (Table 1). Among the 220 participants with RMDs not receiving immunosuppression, hydroxychloroquine, or intravenous immunoglobulin, the rate of anti-RBD titers of 250 U/mL or greater was comparable among BNT162b2 (108/118 [91.5%]) and mRNA-1273 (95/102 [93.1%]) recipients (incidence rate ratio [IRR], 1.02; 95% CI, 0.94-1.10; *P* = .69). However, mRNA-1273 recipients had higher rates of anti-RBD titers of 250 U/mL or greater than BNT162b2 recipients among the 938 patients with RMDs receiving immunosuppression (324/409 [79.2%] vs 320/529 [60.5%]; IRR, 1.30; 95% CI, 1.20-1.43; *P*<.001), 260 SOTRs not receiving mycophenolic acid or mycophenolate mofetil (85/128 [66.4%] vs 59/132 [44.7%]; IRR, 1.56; 95% CI, 1.24-1.96; *P*<.001), and 437 SOTRs receiving mycophenolic acid or mycophenolate mofetil (23/202 [11.4%] vs 10/235 [4.3%]; IRR, 2.62; 95% CI, 1.28-5.37; *P* = 0.01). Similar trends were observed with the other cutoffs (Table 2).

**Table 1.** Demographic Characteristics and Immunosuppressive Regimens of Patients With Rheumatic and Musculoskeletal Diseases and Solid Organ Transplant Recipients Stratified by Vaccine Platform^a^

Characteristic	BNT162b2 (n = 647)	mRNA-1273 (n = 511)	*P* value^b^
**Patients with rheumatic and musculoskeletal diseases**			
Age, median (IQR), y	47 (39-57)	48 (37-60)	.30
Sex^c^			
Female	600 (92.9)	455 (89.2)	.04
Male	47 (7.1)	56 (10.8)	
Race and ethnicity			
White	584 (90.1)	477 (93.3)	.07
Minoritized racial and ethnic groups^d^	63 (9.9)	34 (6.7)	
Diagnosis category			
Inflammatory arthritis^e^	245 (37.9)	206 (40.3)	.95
Systemic lupus erythematosus	119 (18.4)	89 (17.4)	
Sjogren syndrome	32 (4.9)	27 (5.3)	
Myositis	34 (5.3)	25 (4.9)	
Vasculitis^e^	18 (2.8)	9 (1.8)	
Systemic sclerosis	5 (0.8)	5 (1.0)	
Other	10 (1.5)	7 (1.4)	
Overlap connective tissues disease^e^	138 (21.3)	111 (21.7)	
Chronic, noninflammatory condition	46 (7.1)	32 (6.3)	
Immunosuppressive regimen			
Not receiving immunosuppressive therapy	68 (10.5)	54 (10.6)	>.99
Hydroxychloroquine or IVIG monotherapy	118 (18.2)	102 (20.0)	.50
Biologic^f^	326 (50.4)	277 (54.2)	.20
Conventional DMARD^g^	430 (66.5)	326 (63.8)	.34
Glucocorticoid^h^	195 (30.1)	123 (24.1)	.02
Combination therapy^i^	386 (59.7)	295 (57.7)	.51
Total immunosuppressants, median (IQR)	2 (1-3)	2 (1-3)	.12
**Solid organ transplant recipients**			
No. of patients	367	330	NA
Sex^c^			
Female	192 (52.3)	190 (57.6)	.19
Male	175 (47.7)	140 (42.4)	
Race and ethnicity			
White	332 (90.4)	295 (89.3)	.71
Minoritized racial and ethnic groups^d^	35 (9.6)	35 (10.7)	
Age, median (IQR), y	62 (48-69)	62 (49-68)	.89
Transplant category			
Kidney	168 (45.8)	142 (43.0)	.71
Liver	82 (22.3)	74 (22.4)	
Pancreas	4 (1.1)	2 (0.6)	
Lung	44 (12.0)	44 (13.3)	
Heart	45 (12.3)	40 (12.1)	
Intestine	2 (0.5)	0 (0.0)	
Multiorgan	22 (6.0)	28 (8.5)	
Time since transplant at dose 1, median (IQR), y	6.4 (2.6-14.0)	5.8 (2.9-13.5)	.94
Immunosuppressive regimen^j^			
Mycophenolate mofetil or mycophenolic acid	235 (64.0)	202 (61.2)	.48
Sirolimus	39 (10.6)	36 (10.9)	.90
Glucocorticoid^g^	205 (55.9)	192 (58.2)	.54
Antimetabolite^k^	257 (70.0)	231 (70.0)	>.99
Calcineurin inhibitors^l^	329 (89.6)	299 (90.6)	.70
Triple immunosuppressants^m^	131 (35.7)	106 (32.1)	.34
Total No. of immunosuppressants, median (IQR)	2 (2-3)	2 (2-3)	.77

Abbreviations: DMARD, disease-modifying antirheumatic drug; NA, not applicable; RBD, receptor binding domain; RMD, rheumatic and musculoskeletal disease; SOTRs, solid organ transplant recipients.

^a^ Data are expressed as number (percentage) of patients unless otherwise indicated.

^b^ Comparisons between groups were performed using the Wilcoxon rank sum test for continuous variables and the Fisher exact test for categorical variables.

^c^ The denominators for these categories differ from the total sample sizes because 7 participants with RMDs and 5 SOTRs did not respond to the question.

^d^ Minoritized racial and ethnic groups include American Indian or Alaska Native, Arab or Middle Eastern, Asian, Black or African American, multiracial, and Native Hawaiian or other Pacific Islander.

^e^ Inflammatory arthritis includes rheumatoid arthritis, ankylosing spondylitis, psoriatic arthritis, reactive arthritis, or inflammatory bowel disease–associated arthritis. Vasculitis includes polyarteritis nodosa, Bechet syndrome, polymyalgia rheumatica, temporal arteritis, eosinophilic granulomatosis polyangiitis, granulomatous polyangiitis, Henoch-Schoenlein purpura, microscopic polyangiitis, or Takayasu arteritis. Other includes adult-onset Still disease, undifferentiated connective tissue disease, and sarcoidosis. Chronic noninflammatory conditions include osteoarthritis and fibromyalgia.

^f^ Biologic therapy includes abatacept, belimumab, adalimumab, certolizumab, etanercept, golimumab, infliximab, ixekizumab, iecukinumab, ustekinumab, tocilizumab, tofacitinib, upadacitinib, and rituximab.

^g^ Conventional DMARDs include azathioprine, hydroxychloroquine, leflunomide, methotrexate, mycophenolate, sulfasalazine, and tacrolimus.

^h^ Prednisone or prednisone equivalents.

^i^ Combination of conventional DMARDs, biologics, and corticosteroid therapy.

^j^ Patients taking belatacept were excluded.

^k^ Includes azathioprine or mycophenolate.

^l^ Includes tacrolimus and cyclosporine.

^m^ Includes 3 concurrent immunosuppressive agents.

**Table 2.** Anti–Receptor Binding Domain Response to 2-Dose Messenger RNA SARS-CoV-2 Vaccination Stratified by Vaccine Platform

Antibody titer, U/mL^a^	BNT162b2, %	mRNA-1273, %	IRR (95% CI)	*P* value
**Patients with RMDs not receiving immunosuppression (n = 220)** ^b^				
≥50	98.3	95.1	0.97 (0.92-1.02)	.20
≥100	95.8	95.1	0.99 (0.94-1.05)	.81
≥250	91.5	93.1	1.02 (0.94-1.10)	.67
**Patients with RMDs receiving immunosuppression (n = 938)** ^b^				
≥50	75.4	83.1	1.10 (1.03-1.17)	.005
≥100	72.4	82.4	1.13 (1.06-1.21)	<.001
≥250	60.5	79.2	1.30 (1.20-1.42)	<.001
**SOTRs not receiving mycophenolic acid or mycophenolate mofetil (n = 260)** ^c^				
≥50	59.1	78.9	1.38 (1.16-1.64)	<.001
≥100	54.6	77.3	1.47 (1.22-1.77)	<.001
≥250	44.7	66.4	1.56 (1.24-1.96)	<.001
**SOTRs receiving mycophenolic acid or mycophenolate mofetil (n = 437)** ^c^				
≥50	12.8	21.8	1.69 (1.11-2.58)	.02
≥100	7.7	18.8	2.40 (1.42-4.07)	.008
≥250	4.3	11.4	2.62 (1.28-5.37)	.01

Abbreviations: IRR, incidence rate ratio; RMD, rheumatic and musculoskeletal disease; SOTRs, solid organ transplant recipients.

^a^ Roche Elecsys anti–receptor binding domain panimmunoglobulin titer of 0.8 U/mL or higher is considered positive per manufacturer (commercial upper ceiling >250 U/mL, with later expansion to >2500 U/mL). Patients with RMDs not receiving immunosuppression, hydroxychloroquine, or intravenous immunoglobulin had a median titer greater than 250 U/mL (IQR, >250-717.3 U/mL); patients with RMDs receiving immunosuppression had a median titer greater than 250 U/mL (IQR, 126.8-434.6 U/mL); SOTRs not receiving mycophenolic acid or mycophenolate mofetil had a median titer greater than 250 U/mL (IQR, 17.72->250 U/mL); and SOTRs receiving mycophenolic acid or mycophenolate mofetil had a median titer less than 0.8 U/mL (IQR, <0.8-9.44 U/mL).

^b^ Rheumatic and musculoskeletal diseases include inflammatory arthritis, systemic lupus erythematosus, systemic sclerosis, myositis, Sjogren syndrome, systemic vasculitis, and overlap connective tissue disease as well as chronic noninflammatory conditions, including osteoarthritis and fibromyalgia.

^c^ Organ transplants include liver, kidney, pancreas, lung, heart, intestine, and multiple organ transplants.

## Discussion

We observed similar seroresponses to mRNA-1273 and BNT162b2 vaccines in patients not receiving immunosuppression. However, patients receiving immunosuppression had higher mRNA-1273 vs BNT162b2 vaccination seroresponses. Differential immunogenicity was highest in the most immunosuppressed participants (SOTRs receiving mycophenolic acid or mycophenolate mofetil), with a 2.6-fold higher rate of achieving anti-RBD titers of 250 U/mL or greater.

Limitations include self-reporting of SARS-CoV-2 infection and lack of virus neutralization testing. However, this self-report correlated well with prevaccination antibody levels, and a previous study^5^ correlated anti-RBD levels with neutralization capacity in vaccinated individuals.

In conclusion, mRNA-1273 was more likely to induce stronger humoral immunogenicity compared with BNT162b2 in immunosuppressed patients; this effect was more pronounced with greater immunosuppression. These findings suggest that choice of mRNA vaccine platform is important in optimizing immune responses to SARS-CoV-2 vaccination and can help inform strategies for booster doses in high-risk, immunosuppressed populations.
